# Pathological Modification of TDP-43 in Amyotrophic Lateral Sclerosis with SOD1 Mutations

**DOI:** 10.1007/s12035-018-1218-2

**Published:** 2018-07-07

**Authors:** Gye Sun Jeon, Yu-Mi Shim, Do-Yeon Lee, Jun-Soon Kim, MinJin Kang, So Hyun Ahn, Je-Young Shin, Dongho Geum, Yoon Ho Hong, Jung-Joon Sung

**Affiliations:** 1 Department of Neurology, Seoul National University Hospital, Seoul, South Korea; 2 Biomedical Research Institute, Seoul National University Hospital, Seoul, South Korea; 3 Department of Biomedical Sciences, Korea University College of Medicine, Seoul, South Korea; 4 Department of Neurology, Seoul National University Seoul Metropolitan Government Boramae Medical Center, Seoul, South Korea; 5 Neuroscience Research Institute, Seoul National University College of Medicine, Seoul, South Korea

**Keywords:** Amyotrophic lateral sclerosis (ALS), Mutant SOD1, TAR DNA binding protein (TDP-43), Glial cells

## Abstract

Amyotrophic lateral sclerosis (ALS) is a fatal, adult-onset, progressive neurodegenerative disorder with no known cure. Cu/Zn-superoxide dismutase (SOD1) was the first identified protein associated with familial ALS (fALS). Recently, TAR DNA-binding protein 43 (TDP-43) has been found to be a principal component of ubiquitinated cytoplasmic inclusions in neurons and glia in ALS. However, it remains unclear whether these ALS-linked proteins partly have a shared pathogenesis. Here, we determine the association between mutant SOD1 and the modification of TDP-43 and the relationship of pathologic TDP-43 to neuronal cytotoxicity in SOD1 ALS. In this work, using animal model, human tissue, and cell models, we provide the evidence that the association between the TDP-43 modification and the pathogenesis of SOD1 fALS. We demonstrated an age-dependent increase in TDP-43 C-terminal fragments and phosphorylation in motor neurons and glia of SOD1 mice and *SOD1*G85S ALS patient. Cytoplasmic TDP-43 was also observed in iPSC-derived motor neurons from *SOD1*G17S ALS patient. Moreover, we observed that mutant SOD1 interacts with TDP-43 in co-immunoprecipitation assays with G93A hSOD1-transfected cell lines. Mutant SOD1 overexpression led to an increase in TDP-43 modification in the detergent-insoluble fraction in the spinal cord of SOD1 mice and fALS patient. Additionally, we showed cellular apoptosis in response to the interaction of mutant SOD1 and fragment forms of TDP-43. These findings suggest that mutant SOD1 could affect the solubility/insolubility of TDP-43 through physical interactions and the resulting pathological modifications of TDP-43 may be involved in motor neuron death in SOD1 fALS.

**Electronic supplementary material:**

The online version of this article (10.1007/s12035-018-1218-2) contains supplementary material, which is available to authorized users.

## Introduction

Amyotrophic lateral sclerosis (ALS) is a fatal, adult-onset, progressive neurodegenerative disorder with no known cure [1]. Approximately 10% of cases are familial ALS (fALS), and the remaining 90% of ALS cases occur sporadically (sALS). Screening for ALS-associated genes can identify pathogenic mutations in more than 60% of fALS cases. However, because of incomplete penetrance, false paternity, recessive inheritance, or de novo mutations, these pathogenic mutations can only be identified in about 8% of sALS cases [2, 3]. Glutamate toxicity, oxidative stress, protein misfolding, altered axonal transport, mitochondrial dysfunction, and defects in RNA processing have all been implicated in motor neuron death in ALS [1].

The causes of ALS are complicated and currently under investigation. More than 12 pathogenic proteins have been genetically identified, including Cu/Zn-superoxide dismutase (SOD1), TAR DNA-binding protein (TARDBP; TDP-43) [4], FUS [5], and more recently, guanine nucleotide exchange factor (C9ORF72) [6] and Ubiquilin2 (UBQLN2) [7, 8]. Among these, mutations in SOD1, which typically functions in catalyzing the removal of superoxide radicals, have been linked to motor neuron death in up to 20% of patients with fALS. Although these ALS associated genes, including RNA-binding protein (TDP-43), autophagy adaptor (UBQLN2), and a possible C9ORF72, have vastly different cellular functions in the typical clinical phenotype, mutations of these genes can all result in ALS. Moreover, it is rare to find a connection between proteins in the pathological mechanism involved in ALS.

Protein mislocalization and aggregation may play an important role in ALS pathology. The aggregate formation of misfolded SOD1, the first identified protein associated with fALS, is closely associated with ALS pathogenesis. TDP-43 proteins are also mislocalized from their normal nuclear compartment to the cytoplasm resulting in the formation of cytoplasmic aggregations [1, 9, 10] TDP-43 plays a major role in regulating RNA splicing, stability, and transport [11]. However, in pathological neurons, TDP-43 is often found ubiquitinated and fragmented in the cytoplasm and thus, prone to aggregation [12–14]. ALS and frontotemporal lobar degeneration with ubiquitin inclusions (FTLD-TDP) have been classified as TDP-43 proteinopathies because of the hallmark presence of TDP-43 cytoplasmic inclusions [15, 16]. TDP-43 has been identified as a major protein in the inclusion body that is ubiquitinated and phosphorylated in both the sALS and fALS [10], and other common pathological features include cleaved fragments of TDP-43 [10, 17]. The characteristic TDP-43 inclusions are present in almost all cases of ALS, including sALS and fALS [1, 9, 10]. However, it remains controversial whether TDP-43 pathology occurs in SOD1-ALS [17–22]. Results from studies using mutant SOD1 mouse lines have shown that TDP-43 is not redistributed in these mice [20, 22]. Thus, it is generally accepted that patients with SOD1 mutations have different TDP-43 pathology behavior. However, some controversial reports suggest that SOD1-related fALS and sALS have common mechanistic pathways, as few SOD1-related fALS cases exhibit cytoplasmic inclusions composed of TDP-43 [10, 23]. Recently, it has been suggested that there is a potential link between TDP-43 and SOD-1 mutations, previously thought to be pathologically distinct pathways of motor neuron degeneration involved sALS and in fALS. Robertson et al. [20] showed mislocalization of TDP-43 to the cytoplasm and an association with UBIs in two fALS cases with SOD1 mutations. Another study shows that modified TDP-43 may be involved in motor neuron death in the spinal cord of a SOD1G93A-expressing ALS mice [24]. Moreover, overexpression of TDP-43 protein in neurons and oligodendrocyte cells causes progressive motor neuron degeneration in SOD1 G93A transgenic mouse models [25]. Furthermore, wild-type forms of TDP-43 and SOD1 misfolds are found in the inclusions of sALS patients [10, 26–28]. Therefore, there is a possibility that the TDP-43 and SOD1 pathologically distinct pathways are connected. However, largely, the relationship between the TDP-43 modification and the developmental pathogenesis of SOD1 fALS has not yet been elucidated and no reports have verified the entire mechanisms of SOD1 and TDP-43. Therefore, in this study, we have determined the association between mutant SOD1 and TDP-43 in a SOD1 G93A transgenic mice model, ALS cell line model, and in spinal cord tissues. Additionally, we have induced pluripotent stem cell (iPSC)-derived motor neurons from SOD1 fALS patients and the relation of TDP-43 with the mutant SOD1-induced motor neuronal cytotoxicity.

## Results

### Age-Dependent Changes in TDP-43 (C-Terminal Form) Modification Increased in the Insoluble and Soluble Fractions of Spinal Cord in hSOD1^G93A^ Mice

**Fig. 1:**
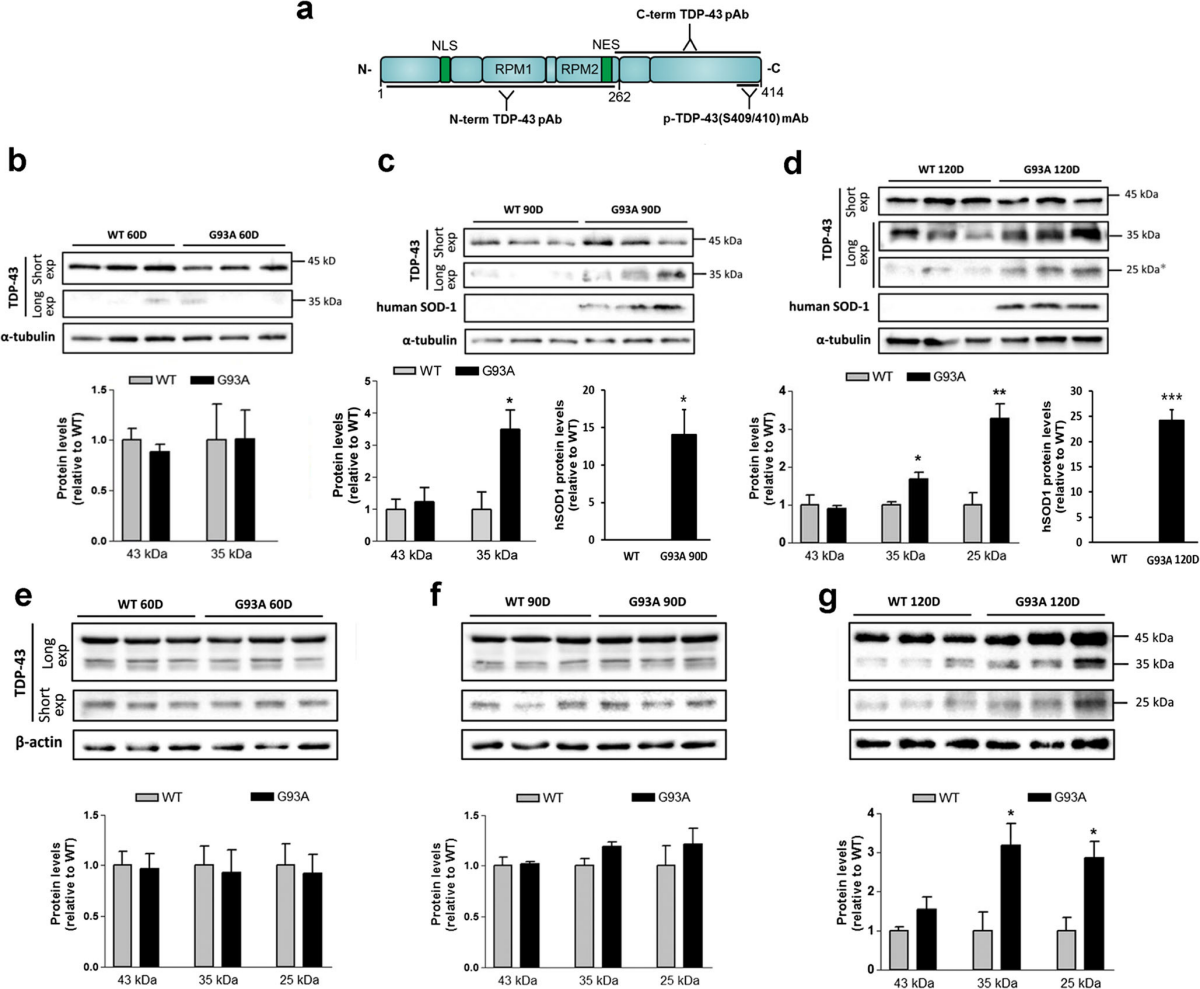
Age-dependent changes in TDP-43 (C-terminal form) modification increased in spinal cord of hSOD1^G93A^ mice. **a** Schematic of selected antibodies mapped to the TDP-43 protein. **b** Age-dependent changes in TDP-43 (C-terminal) modification increased in the soluble and insoluble fraction of spinal cord of hSOD1^G93A^ mice. **b**–**g** Representative western blots of proteins from the insoluble fraction (**b**–**d**) and soluble fraction (**e**–**g**) extracted from 60- (**b**, **e**), 90- (**c**, **f**), and 120-day old (**d**, **g**) hSOD1^G93A^ and WT mice. A longer exposure time was necessary to see the less abundant low molecular weight fragments. **c** In detergent insoluble fraction, the 90-day-old hSOD1^G93A^ mice had significantly higher TDP-35 and human SOD1 levels than WT mice. d. 120-day-old hSOD1^G93A^ mice had significantly higher levels of TDP-35, TDP-25, and human SOD1 than WT mice. **g** At 120-days, the levels of TDP-35 and TDP-25 were significantly increased in hSOD1^G93A^ and WT mice. exp = exposure. Values are mean ± SEM, *n* = 6; *P < 0.05, **P < 0.005, *** *P* < 0.001

Three commercially available antibodies were used. The first antibody has two known epitopes, recognizing both the N-terminal region and the RRM2. The second antibody has an epitope located in the C-terminal region of TDP-43 and the third antibody is located in the C-terminal region and is specific for the phosphorylated serine residues (Fig. 1a). The effect of mutant SOD1 on TDP-43 expression was evaluated throughout disease progression in SOD1^G93A^ ALS mice. We first measured the steady-state levels of TDP-43 and its C-terminal fragments in the insoluble fraction of proteins extracted from 60, 90, and 120-day old SOD1^G93A^ and WT mice (*n* = 6) (Fig. 1b–d). We found that in the lumbar spinal cords of 60-day-old mice, the levels of TDP-43, the ~ 35-kDa C-terminal fragment (herein referred to as TDP-35), and the ~ 25-kDa C-terminal fragment (herein referred to as TDP-25), detected through overexposure of the blots, were similar between SOD1^G93A^ and age- and sex-matched WT mice (Fig. 1b). In contrast, 90-day-old SOD1^G93A^ mice had significantly higher levels of TDP-35 than WT mice (Fig. 1c). Additionally, the 120-day-old SOD1^G93A^ mice had significantly higher levels of TDP-35 and TDP-25 than WT mice (Fig. 1d). Importantly, the amount of insoluble SOD1 was significantly increased in the lumbar spinal cords 90- and 120-day-old SOD1^G93A^ mice (Fig. 1c, d). Subsequently, we measured the levels of TDP-43, TDP-35, and TDP-25 in the soluble fraction of proteins extracted from 60-, 90-, and 120-day-old SOD1^G93A^ and WT mice (*n* = 6) (Fig. 1e–g). We found that in the lumbar spinal cords of 60-days-old mice, the steady-state levels of TDP-43, TDP-35, and TDP-25 were similar between SOD1^G93A^ and WT mice (Fig. 1e). A similar trend was observed in 90-day-old mice (Fig. 1f). However, at the end stage of the disease (120-day-old mice), there was a significant increase in the level of TDP-35 and TDP-25 compared to WT mice (Fig. 1g). The higher levels of TDP-35 and TDP-25 in 120-day-old SOD1^G93A^ mice coincided those in with ALS progression stages [29].

### C-Terminal TDP-43 Protein Immunoreactivity Increased in the Spinal Cords of hSOD1^G93A^ Mice

**Fig. 2:**
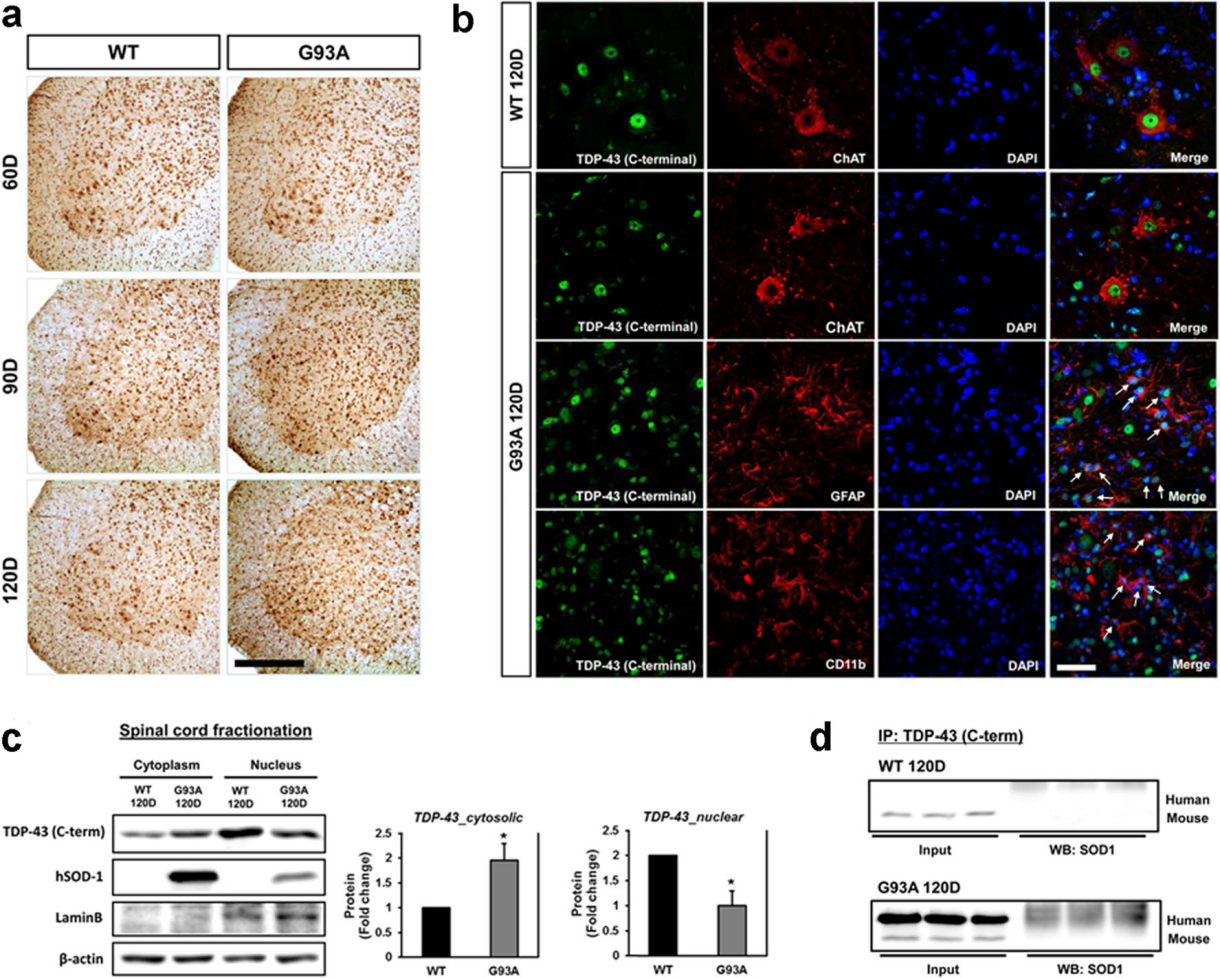
TDP-43 (C-terminal) immunoreactivity increased in the spinal cords of hSOD1^G93A^ mice. **a** Representative images of TDP-43 immunostaining in the ventral horn of lumbar spinal cord sections from WT and hSOD1^G93A^ mice at 60, 90, and 120 days of age. A significant increase in the immunoreactivity of TDP-43 in non-neuronal cell was observed at 90 and 120 days of age in comparison to control mice. **b** TDP-43-positive astrocytes (GFAP^+^) (arrows) or TDP-43-positive microglial cells (CD11b^+^) (arrows). Scale bars: 10 μm. **c** Significantly higher levels of TDP-43 in the cytosolic fraction was observed in 120-day-old hSOD1^G93A^ mice than in the age- and sex-matched WT mice. **d** We confirmed that SOD1-TDP-43 interactions were significantly increased in 120-days-old SOD1^G93A^ mice of lumbar spinal cord. Values are mean ± SEM, *n* = 6; *P < 0.05

We analyzed the distribution of TDP-43 (C-terminal) in the ventral horn of lumbar spinal cord sections from WT littermate control mice and hSOD1^G93A^ mice at 60, 90, and 120 days of age. A significant increase in the immunoreactivity of TDP-43 (C-terminal) in non-neuronal cells was observed in 90- and 120-day-old G93A mice compared to WT control mice (Fig. 2a). TDP-43 (C-terminal) and ChAT were colocalized in motor neurons of 120-day-old WT and G93A mice (Fig. 2b). TDP-43 (C-terminal) immunoreactivity was found mainly in the nuclear compartment of motor neurons in WT mice, whereas in 120-day old hSOD1^G93A^ mice, its expression was elevated in non-neuronal cells in the spinal cord (Fig. 2b). As TDP-43 (C-terminal) appeared to also be localized in non-neuronal cells, we performed double staining using TDP-43 (C-terminal) and GFAP, an astrocyte marker. We found that TDP-43 (C-terminal) and GFAP were colocalized in astrocytes in 120-day-old hSOD1^G93A^ mice. In addition, TDP-43 (C-terminal) and CD11b, a microglia marker, were partially colocalized in some microglial cells of 120-day-old hSOD1^G93A^ mice at 120 days of age (Fig. 2b). To evaluate altered nucleocytoplasmic localization, we prepared fractionated lysates from the spinal cord of 120-day-old WT and hSOD1^G93A^ mice, and western blot was used to measure levels of TDP-43 (C-terminal). The level of TDP-43 (C-terminal) was higher in the spinal cord of hSOD1^G93A^ mice than WT mice in cytoplasmic fractions (Fig. 2c). These results suggest that mutant SOD1 could affect the abnormal mislocalization of TDP-43 in the spinal cord of hSOD1^G93A^ mice. Also, we confirmed that SOD1-TDP-43 interactions were significantly increased in motor neurons of the lumbar spinal cord in hSOD1^G93A^ mice at 120 days of age compared to WT mice (Fig. 2d).

### TDP-43 (N-Terminal) Immunoreactivity and Protein Levels Are Not Altered in the Spinal Cords of hSOD1^G93A^ Mice

**Fig. 3:**
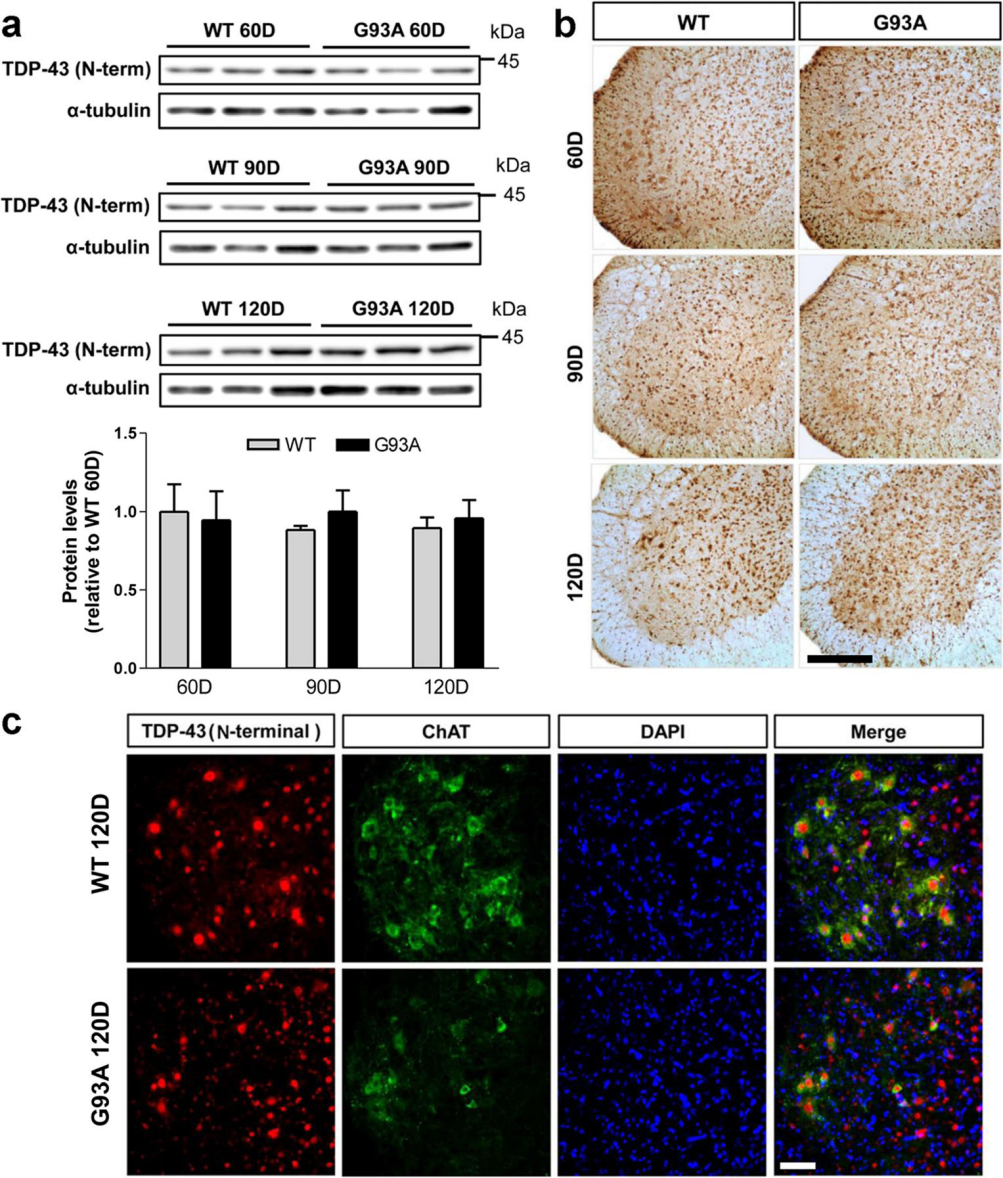
TDP-43 (N-terminal) protein levels and immunoreactivity are not altered in the spinal cords of hSOD1^G93A^ mice. **a** Western blot and densitometric analysis showed constant expression of TDP-43 (N-terminal) in the proteins extracted from 60, 90, and 120 days of age. **b** TDP-43 (N-terminal) staining was seen in the nucleus of spinal motor neuron and interneurons in the spinal cord of WT and hSOD1^G93A^ mice. **c** Note that TDP-43 (N-terminal) and ChAT colocalization in motor neurons was reduced and shown as aberrant patterns in the cytoplasm and nucleus of motor neurons in hSOD1^G93A^ mice. Scale bars 10 μm

Previously, it was found that the N-terminal region of TDP-43 is required for sequestration of full length TDP-43 into cytoplasmic TDP-43 inclusions and subsequent TDP-43 loss-of-function in cultured cells [30]. Therefore, we subsequently measured the steady-state levels of TDP-43 (N-terminal) in the proteins extracted from 60-, 90-, and 120-day-old hSOD1^G93A^ and WT mice (*n* = 6). Western blot and densitometric analysis showed consistent expression of TDP-43 (N-terminal) in the spinal cord of hSOD1^G93A^ mice at 60, 90, and 120 days of age (Fig. 3a). We prepared spinal cord sections from WT and hSOD1^G93A^ mice at 60, 90, and 120 days of age to characterize TDP-43 (N-terminal) immunoreactivity. TDP-43 (N-terminal) staining was seen in the nucleus of spinal motor neurons and interneurons in the spinal cord of WT and hSOD1^G93A^ mice (Fig. 3b). TDP-43 (N-terminal) and ChAT were colocalized in motor neurons both in WT and hSOD1^G93A^ mice at 120 days. At this time point, colocalized motor neurons were reduced and aberrant morphology was observed (Fig. 3c). TDP-43 (N-terminal) immunoreactivity was found mainly in the nuclear compartment of motor neurons in the spinal cord of WT and hSOD1^G93A^ mice at 120 days of age (Fig. 3c).

### The Phosphorylated TDP-43 Protein Levels and Immunoreactivity Increased in the Spinal Cords of hSOD1^G93A^ Mice

**Fig. 4:**
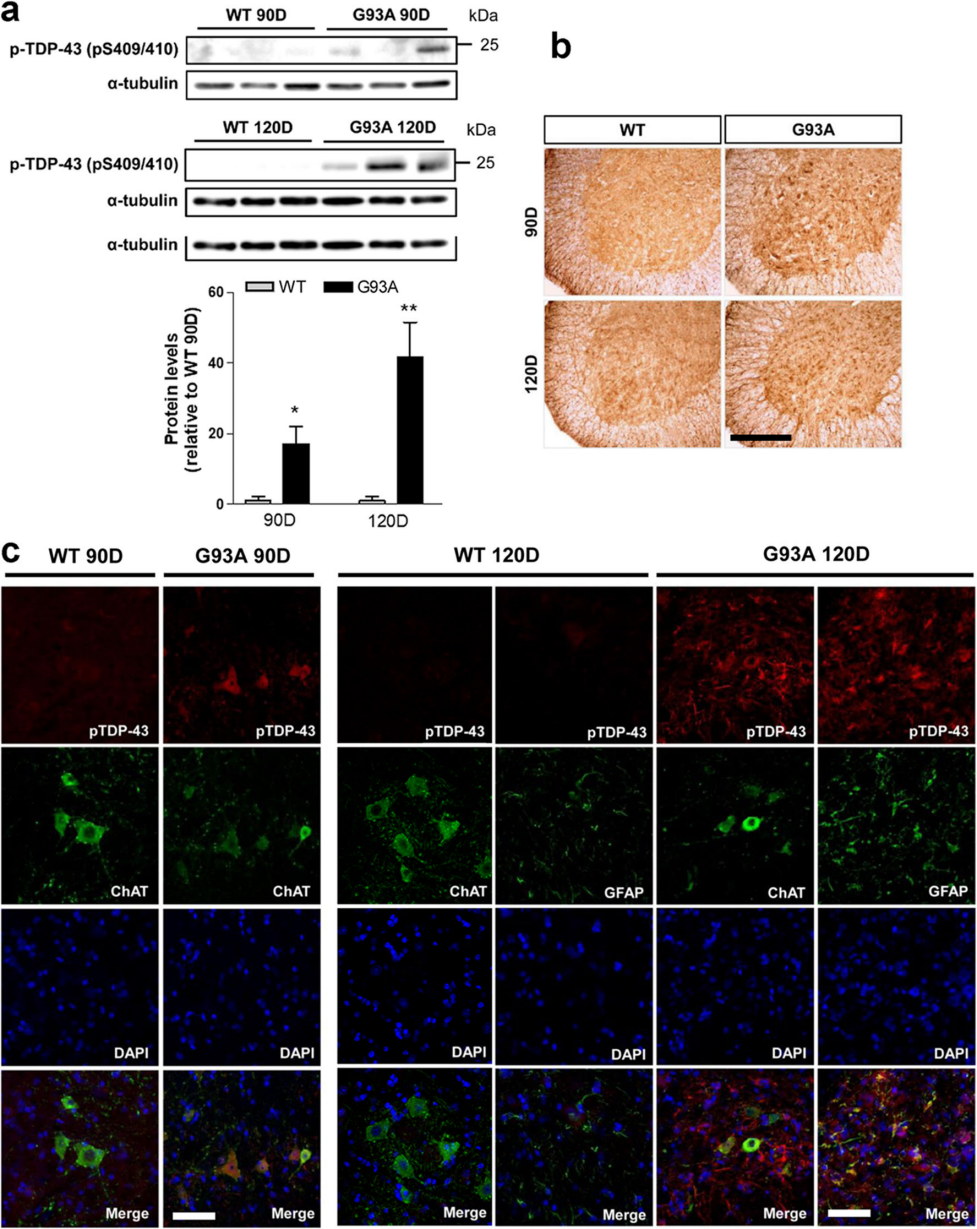
p-TDP-43 protein levels and immunoreactivity are increased in the spinal cords of hSOD1^G93A^ mice. **a** Western blot and densitometric analysis showed that the levels of p-TDP-43 were not detected between SOD1^G93A^ and WT mice at 60-days-old. The levels of p-TDP-43 were only observed in 90-days-old SOD1^G93A^ mice. Moreover, at the end stage of the disease (120-days-old), there was a significant increase in the level of p-TDP-43 in SOD1^G93A^ mice. **b** p-TDP-43 immunoreactivity was not observed in lumbar spinal cord in WT mice and SOD1 ^G93A^ mice at 60 days of age. Motor neurons in lumbar spinal cords from onset stage SOD1 ^G93A^ mice, but not those of WT animals of the same age, showed strong cytoplasmic labeling with p-TDP-43 antibodies. **c** Using double staining for p-TDP-43 and ChAT. p-TDP-43 immunoreactivity was found mainly in ChAT positive cytoplasmic compartment of motor neurons in G93A mice at 90 days of age. A significant increase in the immunoreactivity of p-TDP-43 in non-neuronal cells was observed at 120 days of age in comparison to control mice (**b**). p-TDP-43 and GFAP were colocalized in hSOD1^G93A^ mice at 120 days of age (**c**). Values are mean ± SEM, *n* = 6; *P < 0.05, **P < 0.01. Scale bars 10 μm

To investigate the involvement of p-TDP-43 protein in SOD1 mutant pathology, we measured the levels of p-TDP-43 (pS409/410) in proteins extracted from 60-, 90-, and 120-day-old hSOD1^G93A^ and WT mice (*n* = 6) (Fig. 4a). We found that in the lumbar spinal cords of 60-day-old mice, p-TDP-43 levels were not detected in hSOD1^G93A^ or WT mice (data not shown). At 90 days, p-TDP-43 was observed in SOD1^G93A^ mice, but not in WT mice. At 120 days, there was a significant increase in the level of p-TDP-43 in SOD1^G93A^ mice (Fig. 4a). The higher levels of p-TDP-43 in 90- and 120-day-old hSOD1^G93A^ mice coincided with the ALS progression stages [29]. These results suggest that the mutant SOD1 could increase levels of p-TDP-43 and may be involved in motor neuron death and activated gliosis in hSOD1^G93A^ mice. Subsequently, to more comprehensively evaluate p-TDP-43 pathology, we examined the distribution and cellular localizations of p-TDP-43 from onset (90 days) and progressive (120 days) stage hSOD1^G93A^ mice (Fig. 4b–c). p-TDP-43 immunoreactivity was not observed in the lumbar spinal cord in WT mice or hSOD1 ^G93A^ mice at 60 days of age (data not shown). Motor neurons in lumbar spinal cords from the onset stage of the disease (90-days-old) in hSOD1 ^G93A^ mice showed strong cytoplasmic labeling with p-TDP-43 antibodies compared to WT mice. p-TDP-43 immunoreactivity was found mainly in ChAT positive cytoplasmic mislocalization of motor neurons in hSOD1^G93A^ mice at 90 days of age (Fig. 4b, c). A significant increase in the immunoreactivity of p-TDP-43 in non-neuronal cells was observed at 120 days of age in comparison to WT mice (Fig. 4b). We also found that p-TDP-43 and GFAP were colocalized in astrocytes in hSOD1^G93A^ mice at 120 days of age (Fig. 4b, c).

### Mutant SOD1 Causes Biochemical Alteration of TDP-43

**Fig. 5:**
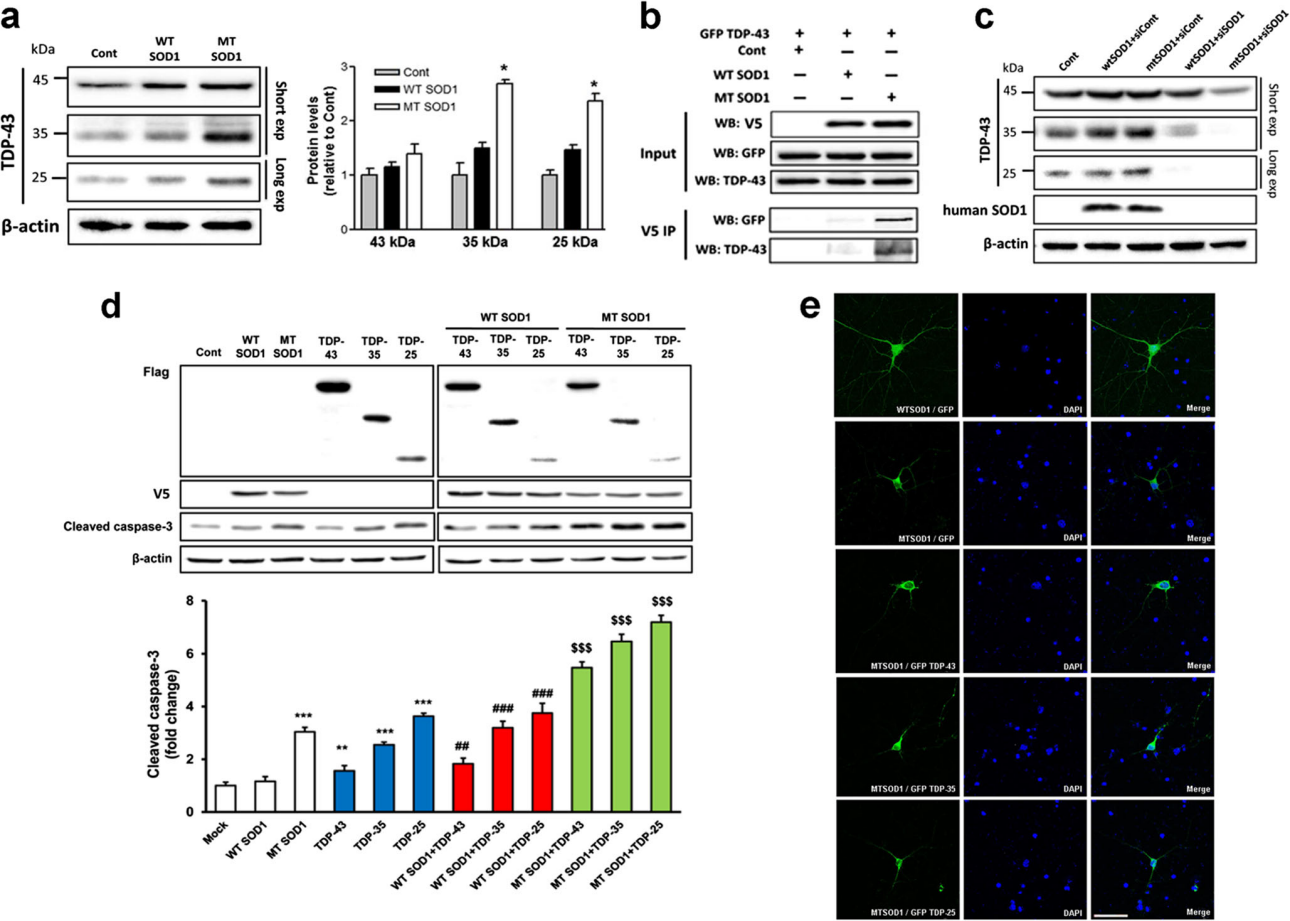
Mutant SOD1 causes biochemical alteration of TDP-43 in NSC-34 cells and primary cortical neurons. **a** NSC-34 cells were transfected with wild-type (WT) SOD1 or mutant (MT) SOD1, and the change in TDP-43 (C-terminal) protein was examined using immunoblotting. MTSOD1 transfected cells had significantly higher level of TDP-35 and TDP-25. **b** Western blots with anti-GFP or anti-TDP-43 showing reciprocal co-immunoprecipitation of SOD1 (anti-V5). We found that SOD1-TDP-43 interactions were significantly increased in MTSOD1 transfected cells. **c** SOD1 small interfering RNA (siRNAs) downregulated the levels of TDP-43, TDP-35, and TDP-25 in MTSOD1 transfected NSC-34 cells. **d** NSC-34 cells were co-transfected with flag-TDP-43, flag-TDP-35, and flag-TDP-25 or V5-WTSOD1 or V5-MTSOD1. Co-transfection with V5-MTSOD1 and flag-TDP-43, flag-TDP-35, or flag-TDP-25 additively increased cleaved caspase-3 levels at 48 h after transfection. **e** WT and G93A-SOD1 mice primary cortical neurons in DIV10 were transfected with GFP or GFP-TDP-43, its fragments GFP-TDP-35 or GFP-TDP-25 for 48 h. TDP-43, TDP-35, and TDP-25 increased G93A-SOD1 mice primary neuronal cell death. Values are mean ± SEM, *n* = 4; *P < 0.05, **P < 0.01, *** *P* < 0.001

Given that both SOD1 and TDP-43 are associated with motor neuron degeneration in patients with ALS, we aimed to determine if mutant SOD1 expression directly causes biochemical alterations of TDP-43 related to motor neuron degeneration in a NSC34 cell line transiently transfected with mutant SOD1. Forty-eight hours after transfection, we detected a significant increase in the levels of cleaved TDP-43 in NSC34 cells transfected with mutant SOD1. Mutant SOD1 transfected cells had significantly higher levels of TDP-35 and TDP-25 than NSC34 cells transfected with mock control or WT SOD1 (Fig. 5a). This finding indicates that mutant SOD1 induces the biochemical alteration of TDP-43 in NSC34 cells. Furthermore, we performed immunoprecipitation of WT or hSOD1^G93A^ mutant SOD1 transfected NSC-34 cell lysates with V5 antibody, followed by western blot analysis of V5 or TDP-43 levels. We found that SOD1–TDP-43 interactions significantly increased in mutant SOD1 transfected cells (Fig. 5b). These results suggest a direct interaction of TDP-43 and mutant SOD1 in NSC34 cells. To investigate the relationship between increased cleaved TDP-43 levels and mutant SOD1, we used RNA interference to knockdown SOD1. We found that SOD1 knockdown partially inhibited the expression of full-length TDP-43 and completely inhibited the TDP-35 and TDP-25 in NSC34 cells transfected with mutant SOD1 (Fig. 5c). Finally, to estimate cellular apoptosis in response to the interaction of mutant SOD1 and the fragment forms of TDP-43, we co-transfected cells with one flag-TDP-43, flag-TDP-35, flag-TDP-25, and one V5-WT SOD1 or V5-mutant SOD1 in NSC34 cells. Forty eight hours after transfection, we observed significantly increased levels of cleaved caspase-3 levels in the groups co-transfected with V5-mutant SOD1 and flag-TDP-43, flag-TDP-35, or flag-TDP-25 compared to transfection with V5-only-mutant SOD1 (Fig. 5d). WT and G93A-SOD1 mice primary cortical neurons in DIV10 were transfected with GFP or GFP-TDP-43, its fragments GFP-TDP-35 or GFP-TDP-25 for 48 h. Under similar conditions, we found that TDP-43, TDP-35, and TDP-25 increased G93A-SOD1 mice primary neuronal cell death (Fig. 5e). Thus, we propose that the synergistic effect of mutant G93A SOD1 and fragment forms of TDP-43 may mediate toxic events in apoptosis.

### Modifications of TDP-43 in the Human Spinal Cord of the *SOD1*G86S Mutant ALS

**Fig. 6:**
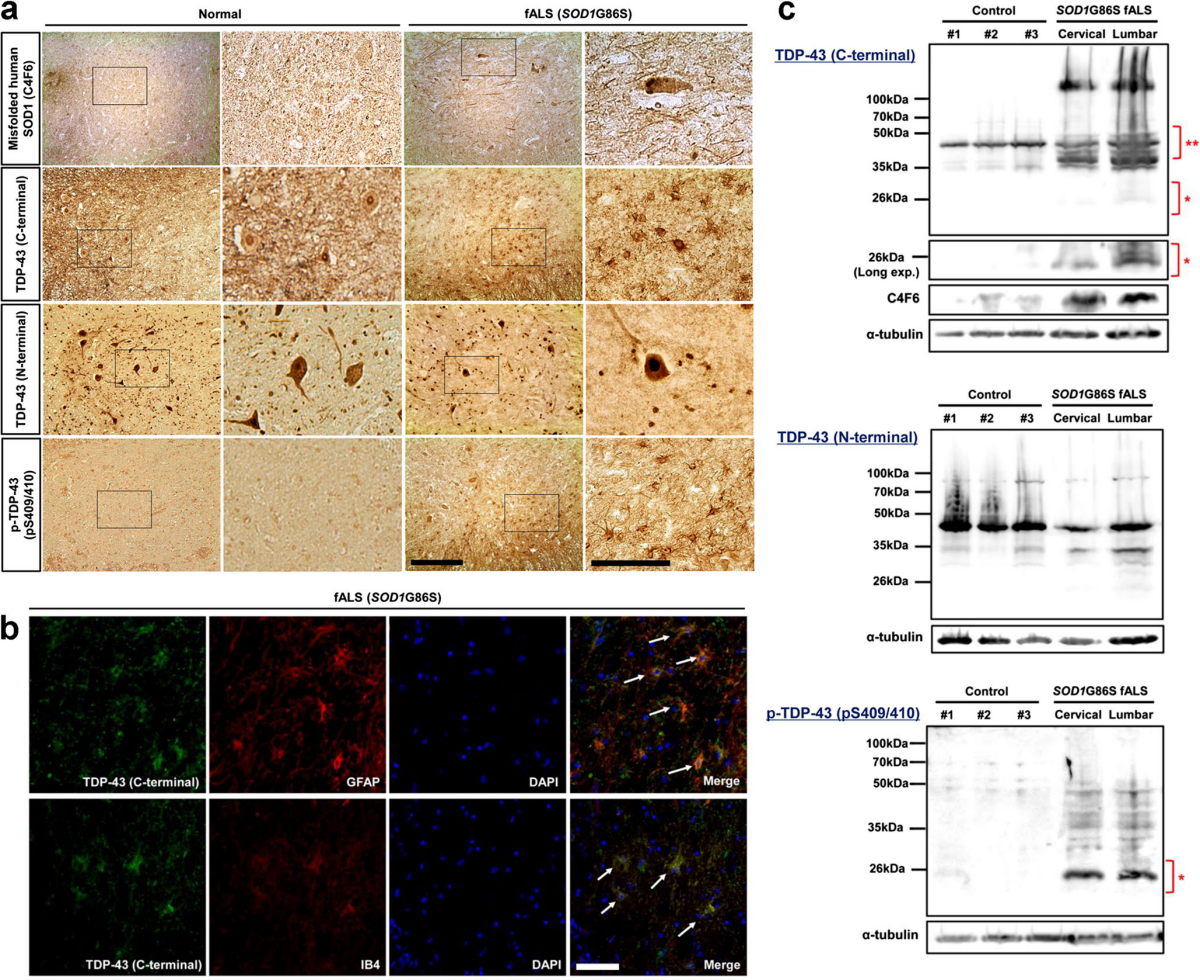
Modifications of TDP-43 in *SOD1*G86S fALS case lumbar spinal cord. **a** Inclusions immunopositive for C4F6, TDP-43 (C-terminal), TDP-43 (N-terminal), and p-TDP-43 were detected in the lumbar spinal cord. Misfolded human SOD1 shows distinct skein-like intracellular inclusions immunoreactive with C4F6 in motor neurons. Nuclei in large neurons in normal control are TDP-43 (C-terminal and N-terminal) positive. Pathologic TDP-43 (C-terminal) and p-TDP-43 immunoreactivity was not found in the lumbar spinal cord of normal control, whereas abundant TDP-43 (C-terminal) and p-TDP-43-positive inclusions were present in glial cells of the lumbar spinal cord (higher magnification of the black box). In contrast, TDP-43 (N-terminal) immunoreactivity was found in the motor neurons and interneurons in the nucleus. **b** Representative micrographs from confocal microscopy using anti-TDP-43 (C-terminal). Double immunofluorescence staining for TDP-43 (C-terminal) and glial fibrillary acidic protein (GFAP; astrocyte marker) or isolectin B4 (IB4; microglial marker) in the lumbar spinal cord of *SOD1*G86S fALS patient. TDP-43 immunoreactivity was colocalized (arrows) with GFAP or IB4 in the lumbar spinal cord of *SOD1*G86S fALS patient. Abundant TDP-43 (C-terminal) and p-TDP-43-positive inclusions were present in glial cells of the lumbar spinal cord. **c** The protein levels of TDP-43 (C-terminal) are increased in ALS. Representative western blots of proteins from the insoluble fraction extracted from control and *SOD1*G86S fALS patient. A longer exposure time was necessary to see the less abundant low molecular weight fragments. Levels of TDP-35 and TDP-25 or p-TDP-43 significantly increased in the cervical (C) and lumbar (L) spinal cord of fALS patient compared to normal controls. Notably, anti-TDP-43 (C-terminal) recognized the pathological C-terminal fragments (*) and 45-kD bands (**) in the urea fraction. Alternatively, p-TDP-43 recognized the pathological bands (*) in the urea fraction, whereas anti-TDP-43 (N-terminal) detected a 45-kD and 35-kD band in the urea fraction. Scale bar 25 μm

We subsequently investigated whether the modifications of TDP-43 observed in hSOD1^G93A mice^ and ALS cell lines were also observed in human spinal cord of an SOD1 mutant ALS case (Table S1). Immunohistochemical analysis was performed with antibodies specific to anti-misfolded SOD1 (C4F6), anti-TDP-43 (C-terminal), anti-TDP-43 (N-terminal), and anti-p-TDP-43 (Fig. 6a). Inclusions immunopositive for C4F6, TDP-43 (C-terminal), and p-TDP-43 were detectable in the lumbar spinal cord of *SOD1*G86S fALS case. Higher levels of mislocalized TDP-43 (C-terminal) compared to TDP-43 (N-terminal) were detected in the lumbar spinal cord. Misfolded human SOD1 showed distinct skein-like intracellular inclusions immunoreactive with C4F6 in motor neurons. Abundant TDP-43 (C-terminal) and p-TDP-43-positive inclusions were present in glial cells of the G86S SOD1 lumbar spinal cord. Double immunofluorescent staining revealed that TDP-43 (C-terminal) colocalized with GFAP or IB4 (Fig. 6b). In contrast, TDP-43 (N-terminal) immunoreactivity was found in motor neurons and interneurons in the nucleus (Fig. 6a). To further biochemical characterization of the TDP-43 protein, we performed immunoblots on urea fractions from the lumbar spinal cord of the *SOD1*G86S fALS case. Notably, anti-TDP-43 (C-terminal) recognized the pathological C-terminal fragments (*) and 45-kD bands (**) in the urea fraction. p-TDP-43 recognized the pathological bands (*) in the urea fraction, whereas anti-TDP-43 (N-terminal) detected no differences in the 45-kD and 35-kD bands in the urea fraction (Fig. 6c). These results suggest that an increase in cleaved TDP-43 and p-TDP-43 levels may be involved in motor neuron death in the spinal cord of SOD1 mutation fALS patients.

### Localization of TDP-43 in iPSC-Derived Motor Neurons from the *SOD1*G17S Familiar ALS Patient

**Fig. 7:**
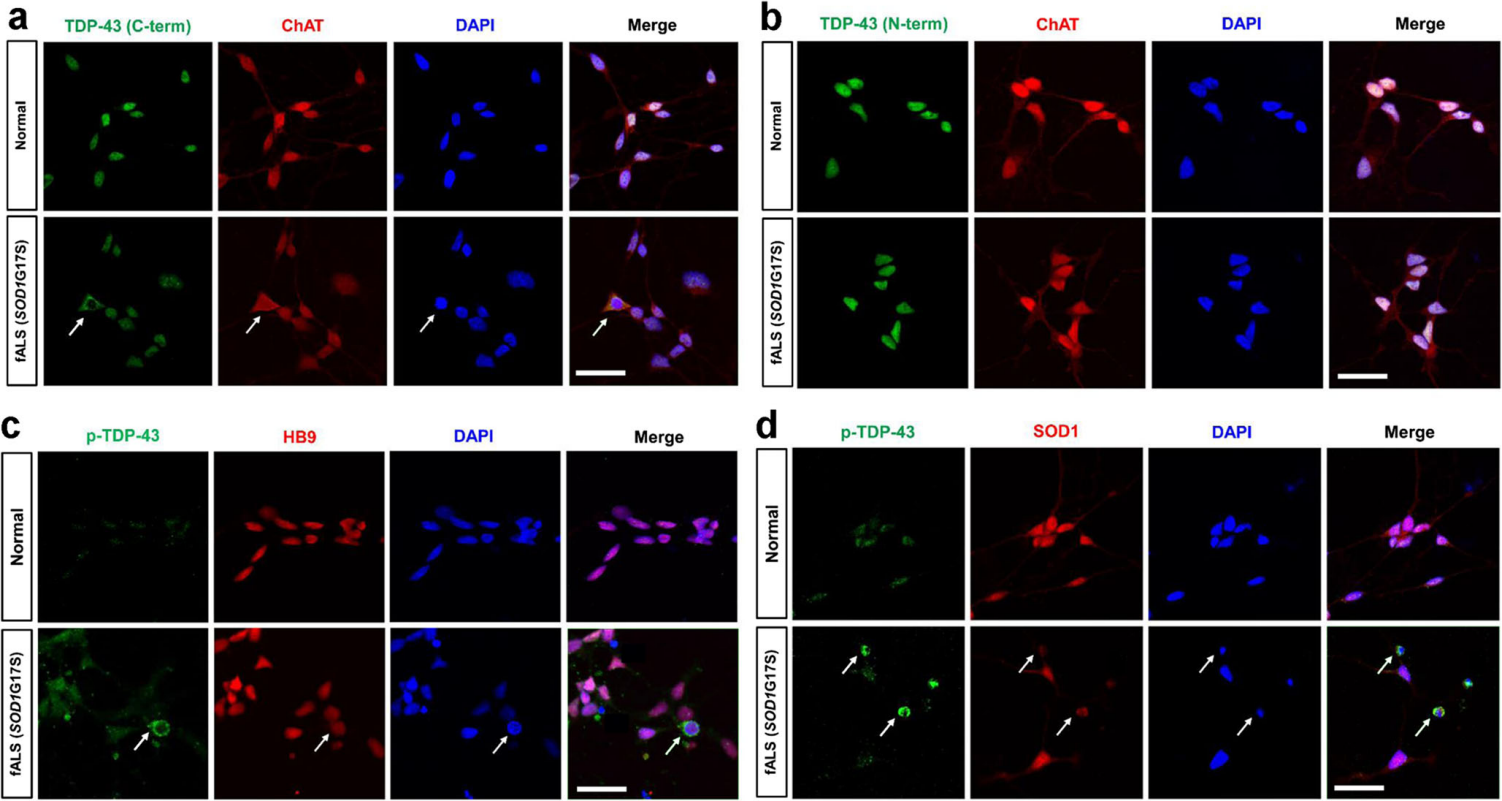
Localization of TDP-43 in iPSCs-derived motor neurons from *SOD1*G17S fALS patient. Representative micrographs at confocal microscopy using three TDP-43 antibodies. **a**, **b** The normal shows intense TDP-43 (C-terminal and N-terminal) staining in all nuclei. **a** Diffuse cytoplasmic mislocalization of TDP-43 (C-terminal) colocalized with ChAT positive motor neurons (arrows). **b** TDP-43 (N-terminal) only localized with nucleus of ChAT positive motor neurons. **c**, **d** p-TDP-43-positive inclusions colocalized with SOD-1-immunoreactive inclusions in the motor neuronal cytoplasm (arrows)

To better confirm pathological TDP-43 in SOD1 familiar ALS, we generated and characterized control and *SOD1*G17S human iPSC lines and differentiated them into motor neurons (Table S1). The control shows intense TDP-43 (C-terminal and N-terminal) staining in all nuclei (Fig. 7a, b), whereas the *SOD1*G17S TDP-43 (C-terminal) was mislocalized in cytoplasm in ChAT positive motor neurons (Fig. 7a). TDP-43 (N-terminal) was localized at the nucleus of ChAT positive motor neurons, similar to controls (Fig. 7b). Double immunofluorescent staining revealed pathologic p-TDP-43 immunoreactivity in cytoplasmic inclusions, and p-TDP-43-positive inclusions colocalized with SOD-1-immunoreactive inclusions in the motor neuron cytoplasm (Fig. 7c, d).

## Discussion

In the present study, we demonstrated that pathological TDP-43 truncation and phosphorylation increased in SOD1 mutation ALS. We have employed as well-characterized and validated antibodies, TDP-43 (C-terminal), TDP-43 (N-terminus), and phosphor (409/410)-TDP43, which detect the truncated and phosphorylated forms of TDP-43 with these in order to evaluate for the modification of TDP-43. Moreover, our results show that abnormal TDP-43 expression aggravated cytotoxicity in SOD1 mutation ALS. We report several lines of novel evidence for the relationship between TDP-43 and SOD1 mutations in ALS.

First, we showed that accumulation of low molecular weight C-terminal fragments of TDP-43 is related to mutant SOD1 pathogenesis. To further confirm this, we showed that in the spinal cords of hSOD1^G93A^ mice, the levels of full length TDP-43 and its ~ 35- and 25-kDa C-terminal fragment changed as a function of age and SOD1 levels in insoluble and soluble fractions. Notably, we found that TDP-43 levels and fragments significantly correlated with insoluble SOD1 levels, suggesting a possible relationship between SOD1 and TDP-43. We also found that mutant SOD1 expression increased the amount of TDP-35 and 25 C-terminal fragments in the cell lysate (Fig. 5a), and that cellular apoptosis increased in response to the interaction of mutant SOD1 and fragment forms of TDP-43 when we co-transfected with one of flag-TDP-43, flag-TDP-35, and flag-TDP-25 and one of GFP-WT SOD1 or GFP-mutant SOD1 in NSC34 cells (Fig. 5d). Two days after transfection, we observed that cleaved caspase-3 expression significantly increased in the groups co-transfected with GFP-mutant SOD1 and flag-TDP-35, or flag-TDP-25 in comparison to the transfection with GFP-mutant SOD1 and flag-TDP-43 (Fig. 5d). Thus, we propose that a synergistic effect of mutant SOD1 and fragment forms of TDP-43 mediate toxic events in apoptosis. Furthermore, mutant SOD1 fALS patient spinal cord showed accumulation of C-terminal fragments in the urea insoluble fraction. The mechanism for how TDP-43 fragments are produced because of SOD1 mutations is unclear. Typically, TDP-43 C-terminal fragments are suggested to be generated through proteolytic cleavage of full-length TDP-43 secondary to its abnormal cytoplasmic mislocalization in glial cells [31]. Furthermore, it has been suggested that splice variants or cryptic translation start sites may contribute to the production of TDP-43 CTFs in ALS [32]. However, this does not explain the increase in TDP-43 fragments among various cellular components that we observed. The mechanism of generation of TDP-43 toxic fragments requires further study; however, we observed a direct interaction between mutant SOD1 and TDP-43 in an ALS cell line (Fig. 5b) and the spinal cord of the hSOD1^G93A^ mice at 120 days of age (Fig. 2d) via co-immunoprecipitation. Additionally, a previous study showed that a mutant SOD1 interacts with TDP-43 using co-immunoprecipitation assays in cultured cells and SOD1^G93A^ transgenic mice. The region responsible for this interaction within SOD1 is the dimer interface, namely, the N- and C-terminal regions. They proposed that aberrant interactions between the dimer interface of mutant SOD1 and TDP-43 could underlie a gain of toxic functions of mutant SOD1 [33].

Second, we confirmed TDP-43 pathology in various models of SOD1 mutation ALS. Robertson et al. [20] found inconsistencies between human and transgenic mouse samples and thus was unable to provide strong evidence of a pathological mechanism. In contrast to previous reports, we observed redistribution of TDP-43 to the cytoplasm of motor neurons in hSOD1^G93A^ mice. Subcellular fractionation indicated that hSOD1^G93A^ mice had significantly higher levels of TDP-43 in the cytosolic fraction than WT mice (Supplemental Fig. 1). Despite finding pathological TDP-43 relocalization, Shan et al. [21] failed to observe C-terminal TDP-43 fragments (~ 25 kDa) and TDP-43 hyperphosphorylated species (~ 45 kDa) in end-stage mutant SOD1 mice [21]. Notably, we found that TDP-43 and its ~ 35- and 25-kDa C-terminal fragments (CTFs) levels were higher from onset stages (90 days) of hSOD1^G93A^ mice compared to WT mice in the insoluble fraction. We also found that TDP-43 and its fragments form levels significantly correlated with insoluble SOD1, suggesting a possible relationship between SOD1 and TDP-43. This result is not merely coincidental seen in the end stages, suggesting that they are involved in SOD1 disease progression. Although further studies are warranted to elucidate the mechanisms and the pathogenic relevance of interactions between SOD1 and TDP-43, our findings clearly indicate that SOD1 mutations alter TDP-43 metabolism. Moreover, our results show the abnormal TDP-43 expression is consistently observed in the hSOD1^G93A^ ALS cell line model, the spinal cord tissues, and iPSCs-derived motor neurons from fALS patients with *SOD1* G86S and G17S mutations, respectively. An association between misfolded and/or aggregated SOD1 and TDP-43 mislocalization has been previously suggested. For example, a previous study reported that TDP-43 cytoplasmic mislocalization and deposition into ubiquitin immunoreactive inclusions were observed in lower motor neurons of end-stage mutant SOD1 transgenic mice [21]. In addition, misfolded human WT SOD1 has been associated with cytosolic accumulation of mutant TDP-43 in TDP-43-fALS and WT TDP-43 in sALS. It has also been suggested that mutant TDP-43 may indirectly induce the propagation of WT SOD1 misfolding in fALS and sALS [34]. Our results confirm and extend the results of these previous studies using nearly every model of ALS from cell lines, to human tissue and iPSC. The fact that SOD1 mutation patients with other different mutations exhibited the same pattern of TDP-43 abnormality supports the hypothesis that TDP-43 abnormality has a role in SOD1 mutation pathogenesis. We provide evidence that a direct interaction between SOD1 and TDP-43 may occur in motor neuronal death. SOD1 has been reported to mainly be a cytosolic protein; however, it is also present in the nucleus [35]. TDP-43 is primarily a nuclear protein, but its presence in the cytoplasm has been well documented [36, 37]. In this study, we observed that mutant SOD1 was localized mainly in the cytosol, whereas endogenous TDP-43 was detected in the cytosolic fraction as well as the nuclear fraction (Fig. 2c). These results suggest that the interaction between mutant SOD1 and TDP-43 may occur in the cytoplasm. Accordingly, our spinal cord tissue and iPSC derived motor neurons from fALS patients showed cytoplasmic mislocalization of truncated and phosphorylated TDP-43.

Third, we observed all pathological changes of TDP-43 (truncation, phosphorylation, mislocalization, and cytoplasmic inclusion) and showed that it was caused by SOD1 mutation. Pathological TDP-43 accumulates as insoluble aggregates in neurons and glia of patients with ALS [10]. Studies on ALS patients have consistently shown that in addition to cytoplasmic accumulation, TDP-43 undergoes posttranslational modifications, including hyperphosphorylation, ubiquitination, and cleavage into small CTFs [10, 17, 18, 23, 38–40]. The accumulation of phosphorylated CTFs is an important finding as it is more abundant and involves additional cells than full-length TDP-43 [40]. Several authors suggest that CTFs significantly contribute to the pathogenesis of TDP-43 proteinopathy [14, 41, 42]. In the present study, we found that TDP-43 CTFs in SOD1 mutation ALS are relatively insoluble and aggregation-prone, similar to recent results from yeast and sALS spinal cords and brains [12, 43]. These findings show that the generation of CTFs is sufficient to initiate a number of events including, cytoplasmic localization, ubiquitination, phosphorylation, and aggregation of TDP-43 CTFs that mirror TDP-43 proteinopathy. Genetically, the C-terminal region of TDP-43 has also been implicated in pathogenesis. The discovery of multiple ALS-associated *TARDBP* mutations that map almost exclusively to the C-terminal domain constitutes an intriguing reminder of the potential link between abnormal TDP-43 post-translational modification, localization, or conformation and the pathogenesis of TDP-43 proteinopathies [4, 44–46]. Interestingly, several of these reported mutations may create novel phosphorylation sites through serine substitution, which may provide the basis for the abnormal properties of mutated TDP-43. However, the availability of more human neuropathological material from *TARDBP* mutation patients is required to test this hypothesis. Further studies are needed to elucidate causal mechanisms underlying the change of TDP-43 pathogenesis. It is possible that the TDP-43 abnormality in SOD1 ALS is produced by a mechanism different from the previously suggested mechanism that the depletion of essential cellular components by mutant TDP-43 induces TDP-43 abnormality. Sabatelli et al., [47] observed TDP-43 pathology in fibroblasts in various cases of fALS and found that TDP-43 protein and mRNA levels were reduced in SOD1 mutation ALS compared to the C9orf72 and TDP 43 mutations. This may suggest that TDP-43 abnormalities were not caused by abnormal turnover of TDP-43, but were instead caused by a different mechanism [47].

Previous studies only observed the TDP-43 protein distribution in the neuronal cell in the ventral horn of spinal cord in the SOD1 wild and G93A transgenic rodents [22, 41], whereas they did not observe the pathological TDP-43 protein expression and altered distribution in the non-neuronal cell types. This is supported by the finding that two patients with different SOD1 mutations exhibited the same peculiar pattern of TDP-43 abnormality. Therefore, the redistribution of TDP-43 protein from the nucleus to the cytoplasm in the spinal motor neurons and glial cells might be characteristic of general ALS pathology [48]. However, previous investigations did not systemically observe TDP-43 protein expression in the spinal motor neurons and glial cells or attempt to determine the type of glial cell [48]. Our result clarified that TDP-43 protein was expressed and redistributed in astrocytes and microglial cells. The increase of TDP-43 protein expression, abnormal distribution in glial cells, negative correlation between the number of glial cells, and the amount of neuron cells in the spinal cord of SOD1 G93A transgenic mice, which was followed by an increase of neuron death (Figs. 2b and 6a, b), implies that overexpression of TDP-43 protein in both neurons and glial cells are a potential non-autonomous mechanism underlying the toxic factor of motor neuron degeneration in ALS. Particularly, histochemical and biochemical analysis showed that the accumulation of truncated and phosphorylated species was consistently observed in our SOD1 G93A transgenic mice. This finding is of interest as only antibodies directed against the C-terminal TDP-43 and p-TDP-43 (phosphorylated serine 409/410) recognize the pathologically altered protein, which label abnormal inclusions [49, 50]. However, further studies in larger number of *SOD1* mutation fALS patients are needed to establish whether p-TDP-43 serves as a pathogenic mediator for SOD1 ALS. In addition, this study also revealed that the expression and redistribution of TDP-43 protein in the neuron and glial cells significantly increased in ALS mice was followed by significant neuron death. Therefore, we suggest that the overexpression of TDP-43 protein in neuron and glial cells could induce the degenerative death of motor neuron in ALS through the pathogenic effect of TDP-43 protein resulting from the formation of toxic aggregates, rather than from the loss of function.

Our findings that TDP-43 modifications (truncation and phosphorylation) increased and the modified TDP-43 was localized abnormally in the cytoplasm of neurons, astrocytes, and microglial cells in symptomatic hSOD1 G93A transgenic mice, an *SOD1*G85S fALS patient and iPSC-derived motor neurons from *SOD1*G17S the fALS patient, recapitulate all hallmarks of TDP-43 abnormalities observed in sALS. Our results clearly indicate that SOD1 mutations alter TDP-43 modification and thus strongly support the hypothesis of an interaction between TDP-43 and mutant SOD1 in ALS pathogenesis. These findings also suggest that mutant SOD1 could affect the solubility/insolubility of its interaction with TDP-43 through physical interaction or an unknown mediator. Further studies are required to clarify the mechanism of TDP-43 modification induced via SOD1 mutation.

## Materials and Methods

### Animals

Transgenic mice expressing the human G93A-mutated SOD1 gene (B6SJL-Tg (SOD1-G93A) 1 Gur/J; Jackson Laboratory, Bar Harbor, Me, USA) were used [29]. Male transgenic ALS mice of the mtSOD1 (G93A) H1 high expressor strain (Jackson Laboratories, Bar Harbor, ME, USA) were bred with female mice with a similar background (B6/SJLF1). The stages of ALS-like disease were divided into three stages according to time point: pre-onset (60–70 days), onset (90–100 days), and progressive (120–130 days). Twelve SOD1 mutant transgenic and ten wild-type mice were used for these experiments at different time points.

### Cell Culture

Motor neuron-like cells (NSC-34) transfected with pCI-neo expression vector containing human wild-type (WT), hSOD1wt (NSC-34/hSOD1wt cells), and mutant hSOD1G93A (NSC-34/hSOD1G93A cells) were established previously [51]. Primary cortical neuron cultures were prepared as previously described [52, 53] briefly, cortical neurons from WT and SOD1-G93A mice were isolated from pups at E16.5, and the brains were removed and placed on ice in Hank’s balanced salt solution (HBSS, Gibco). Twenty-four well plates were seeded with 1 ml of plating medium (2% B27 + 1/100 penicillin/streptomycin, Invitrogen; 0.5-mM α-glutamine; neurobasal medium, Gibco). From DIV4, feed every 3–4 days by removing one half the medium and replacing with fresh.

### Transient Transfection and Small-Interfering RNA Experiment

NSC-34 cells were transfected using LipofectAMINE 2000 reagent (Invitrogen) following the manufacturer’s instructions. NSC-34 cells were transiently transfected with green fluorescent protein (GFP)-wtSOD1 or mtSOD1 and V5-tagged wtSOD1 and mtSOD1, GFP-tagged TDP-43, Flag-tagged TDP-43, TDP-35, and TDP-25 (Flag-tagged TDP-43, TDP-35, and TDP-25 vector, provided by Hong-Yu Hu, Chinese Academy of Sciences, Shanghai, China). NSC-34 cells treated with the pcN1 vector alone were used as negative controls. Transfection efficiency was tested with a vector containing the GFP gene. NSC-34 cells were transiently transfected with 50 nM of stealth control RNAi, SOD-1 siRNA (GenePharma) using Lipofectamine RNAiMAX (Invitrogen Life Tech). Transfection was performed in medium containing 10% FBS without antibiotics. Primary cortical neurons transfected with GFP, GFP-tagged TDP-43, TDP-35, and TDP-25.

### Human Spinal Cord Samples

Post-mortem spinal cord specimens from six normal controls and one with fALS (G86S) were used. Control spinal cord samples were obtained from The Netherlands Brain Bank and the guidelines by The Netherlands Brain Bank were followed. Detailed information of spinal cord tissue is described in Supplementary Table S1. Post-mortem spinal cord specimens from fALS (G86S) spinal cord and blood samples from fALS (G17S) were analyzed with Institutional permission under Review Board in Seoul National University Hospital.

### Histopathological Evaluation (Histology)

For immunohistochemical analysis, mice were perfused with 4% paraformaldehyde (PFA) and the lumbar spinal cords were isolated. Following post-fixation in 4% PFA overnight, spinal cords were cryoprotected in 30% sucrose until the spinal cords sank and then sectioned serially (40 μm). Spinal cord was obtained at autopsy from patients with clinically and pathologically definite ALS and control patients who died of non-neurological diseases. Mouse and human lumbar spinal cord tissue sections were immunostained for TDP-43 (C-terminal), (Proteintech, Rosemont, IL, USA; dilution 1:600), TDP-43 (Proteintech, Rosemont, IL, USA; dilution 1:1000), phosphor TDP-43 (pS409/410) (Cosmo Bio Co., Tokyo, Japan; dilution 1:200), and misfolded human SOD1 (C4F6) (MediMabs, Montréal, Québec, Canada; dilution 1:200) using a previously reported conjugated secondary antibody method in spinal cord tissue samples [51, 54]. Preabsorption with excess target proteins, omission of the primary antibodies, and omission of secondary antibodies were performed to determine the amount of background generated from the detection assay. Immunostained spinal cord sections were observed under a light microscope.

### Immunofluorescence Staining and Confocal Microscopy

Indirect labeling methods were used to identify TDP-43 (C-terminal) (Proteintech, Rosemont, IL, USA; dilution 1:600), TDP-43 (Proteintech, Rosemont, IL, USA; dilution 1:1000), phosphorylated TDP-43 (pS409/410) (Cosmo Bio Co., Tokyo, Japan; dilution 1:200), and misfolded human SOD1 (C4F6) (MediMabs, Montréal, Québec, Canada; dilution 1:200), GFAP, choline acetyltransferase (ChAT), Iba-1, CD11b, IB4, and HB9 in iPSC-derived motor neurons. Motor neurons from the ventral horn of the lumbar spinal cord were stained for choline acetyltransferase (anti-ChAT; Millipore, MA, USA) as previously described [28, 52]. For confocal microscopy, the specimens were incubated for 1 h with DyLight 594 donkey anti-rabbit IgG antibody, DyLight 488 donkey anti-goat IgG antibody, and DyLight 488 donkey anti-mouse IgG antibody (Jackson ImmunoResearch, Baltimore Pike, PA, USA; 1:400) after the incubation of primary antibody. Images were analyzed using a Leica TCS SP8 confocal microscope (Leica Microsystems, Wetzlar, Germany). Preabsorption with excess target protein or omission of primary antibody was used to show antibody specificity and background generated from the detection assay.

### Subcellular Fractionation

We used the commercially available NE-PER nuclear and cytoplasmic extraction reagent (Thermo Scientific, USA). Briefly, the wild-type and mutant NSC-34 cell pellets were washed with phosphate-buffered saline (PBS) and separated through centrifugation. The supernatant was discarded, and the pellet was resuspended in cytoplasmic extraction reagent (CER) I and vigorously vortexed. Subsequently, CER II was added, and the tube was vortexed for 10 s and then centrifuged for 4 min at 16,000 ×*g*. The supernatant was transferred as a cytoplasmic extraction. The insoluble fraction that contained the nuclear component was suspended in ice-cold nuclear extraction reagent (NER) and vortexed for 15 s every 10 min for a total of 40 min. After centrifugation, the supernatant was prepared for the nuclear fraction. The extraction was stored at − 80 °C until use.

### Protein Extraction and Western Blot Analysis

The spinal cord from G93A transgenic mice were isolated, immediately frozen in a deep freezer (− 80 °C), as previously described [55, 56]. Cells cultured in a six-well plate were washed and harvested in PBS using a cell scraper. Protein extracts were isolated by RIPA buffer (Thermo, MA, USA) with protease inhibitors and phosphatase inhibitors added to the buffer (Roche, IN, USA). Each 30-mg protein sample was loaded on an SDS-PAGE gel and transferred onto a PVDF membrane. The blots were probed with primary antibodies for the following: β-actin (1:5000; Sigma, MO, USA), α-tubulin (1:2000; Santa Cruz, CA, USA), TDP-43 (C-terminal), TDP-43 (1:2000; Proteintech, Rosemont, IL, USA), phosphorylated TDP-43 (pS409/410) (1:1000; Cosmo Bio Co., Tokyo, Japan), and misfolded human SOD1 (C4F6) (MediMabs, Montréal, Québec, Canada; dilution 1:200), human SOD1 (1:1000; Cell Signaling), V5 (1:1000; Thermo, MA, USA), Flag (1:1000; Sigma, MO, USA), GFP (1:5000; Rockland, Limerick, PA, USA), and cleaved caspase-3 (1:1000; Cell Signaling). Subsequently, the blots were treated with horseradish peroxidase-conjugated secondary antibodies (anti-mouse, rabbit or goat, 1:5000; GE Healthcare) and imaged using a chemiluminescent kit (Thermo, MA, USA). Membranes were then probed for β-actin or α-tubulin as a loading control. The densitometry of protein intensity was performed using an image analyzer (LAS-3000; Fuji, Tokyo, Japan).

### Immunoprecipitation Analysis

Cell pellets were suspended in lysis buffer containing protease inhibitors, and lysates were centrifuged at 14,000 rpm for 10 min. Equal amounts of protein were precipitated with V5 antibody at 4 °C for 6 h on a rocker. Protein A/G agarose beads (Santa Cruz, CA, USA) were added to each sample and incubated at 4 °C for 1 h. Immunoprecipitates were collected through centrifugation and then washed three times with the same buffer. The agarose beads were resuspended in 30 ml of sample buffer and incubated at 100 °C for 10 min to release the proteins. After a pulse spin, the supernatants were loaded.

### Motor-Neuron Differentiation

Human-induced pluripotent stem cells (hiPSC) were maintained on Matrigel (Thermo Fisher Scientific, USA)-coated culture dishes in mTeSR™1 (Stemcell Technology, Canada) medium. For differentiation, hiPSC colonies were detached using a 2 U/ml Dispase (Thermo Fisher Scientific, USA) for 30 min at 37 °C. Detached colonies were maintained in STEMdiff™ neural induction medium (Stemcell Technology, Canada) for embryoid body (hEBs) formation for 14 days. From day 1 to day 3, hEBs were treated with 3-μM dorsomorphin dihydrochloride (Abcam, USA) and 3-μM SB431542 (Sigma-Aldrich, USA) for neural induction. From day 4 to day 14, hEBs were treated with 1-μM retinoic acid (RA) (Sigma-Aldrich, USA) and 1-mg/ml Sonic Hedgehog (ELPIS-Biotech. Inc., Korea) for motor neuron induction. On day 14, hEBs were enzymatically dissociated into single cells using 0.05% trypsin (Thermo Fisher Scientific, USA). The dissociated cells were plated on poly-L-ornithine (Sigma-Aldrich, USA) and laminin (Sigma-Aldrich, USA)-coated dishes at a density of 5 × 10^4^ cells/cm^2^ and cultured in DMEM F/12 (Thermo Fisher Scientific, USA) with 1% N2 supplement (Thermo Fisher Scientific, USA), 2% B27 supplement (Thermo Fisher Scientific, USA), 1% NEAA (Thermo Fisher Scientific, USA), 0.1-mM 2-ME (Thermo Fisher Scientific, USA), 1-μM RA, 1-mg/ml SHH, 10-ng/ml recombinant BDNF (R&D systems, USA), 10-ng/ml recombinant GDNF (R&D systems, USA), and 10-ng/ml recombinant human NT3 (ELPIS-Biotech. Inc., Korea) for 2 weeks. Half of the medium was changed every day.

### Statistical Analyses

All data are presented as the mean ± standard deviations. Statistical analysis was performed using a Student’s *t* test and one-way ANOVA followed by Fisher’s least significant difference post hoc test. *P* values less than 0.05 were considered statistically significant. Probabilities of symptom onset, rotarod failure, and disease endpoint were analyzed using Kaplan-Meier curves.

## Electronic Supplementary Material

ESM 1 (DOCX 12 kb)
