# Supplementary material for: Pathological Modification of TDP-43 in Amyotrophic Lateral Sclerosis with SOD1 Mutations
Source: Mol Neurobiol. 2018 Jul 7;56(3):2007–21. doi: 10.1007/s12035-018-1218-2 (PMC6394608; doi:10.1007/s12035-018-1218-2)
Supplement: Supplementary file 1 — (DOCX 12 kb) [file 12035_2018_1218_MOESM1_ESM.docx]

**Table 1.** The information for human spinal cord samples

| **Subjects** | **Diagnosis** | **Spinal cord region** | **Age at time of death (yr)** | **Gender** | **Methods** |
| --- | --- | --- | --- | --- | --- |
| Control 1 | Non-demented control | Cervical | 73 | F | Western blot |
| Control 2 | Non-demented control | Cervical | 66 | M | Western blot |
| Control 3 | Non-demented control | Cervical | 73 | F | Western blot |
| Control 4 | Non-demented control | Cervical | 55 | M | Immunohistochemistry |
| Control 5 | Non-demented control | Cervical | 70 | F | Immunohistochemistry |
| Patient 1 | ALS (c.256G>A, p.Gly86Ser, heterozygote) | Cervical, Lumbar | 57 | M | Western blot, Immunohistochemistry |
| Patient 2 | ALS (c.49G>A, p.Gly17Ser, heterozygote) | Blood | 60 | M | iPSCs-derived motor neuron |
